# Multi-model assessments to characterize occurrences of emerald ash borer (Coleoptera: Buprestidae)

**DOI:** 10.1093/jisesa/ieaf032

**Published:** 2025-06-03

**Authors:** Kishan R Sambaraju, Kathryn A Powell, André Beaudoin

**Affiliations:** Natural Resources Canada, Canadian Forest Service, Laurentian Forestry Centre, Québec, QC, Canada; Government of Yukon, Whitehorse, Yukon, Canada; Natural Resources Canada, Canadian Forest Service, Laurentian Forestry Centre, Québec, QC, Canada

**Keywords:** emerald ash borer, predictive modeling, infestation risk, invasion, climate

## Abstract

Introduction and spread of nonindigenous species present a formidable threat to forest health. The emerald ash borer (EAB), *Agrilus planipennis*, is an East Asian-origin insect that has devastated ash (*Fraxinus* spp.) trees across the United States and parts of Canada since 2002. Proactive surveillance using high-performing predictive models could aid in mitigating pest risk. Predictor variables and modeling methods are important considerations in such analysis. Therefore, we assessed whether relevant single predictors, a combination of predictors grouped under a certain driver category, or multiple key predictors comprising several drivers, alter the goodness-of-fit of logistic regression models to EAB occurrence data (2002 to 2018) from Canada. The predictors used in models included spatial, topographic/positional, transport pathways/human hotspots, host-related factors, and climate-related variables. Using predictors from the best candidate logistic regression model, we tested the performance of 7 different model types including an ensemble model. Our findings showed that predictors from a wide range of drivers better characterized EAB occurrences than single predictors or a combination of predictors from any given driver category. In multi-model comparisons, random forest outperformed all other models, including the ensemble model. Elevation, infestation pressure, accumulated degree-days (>10 °C), and human population density were important predictors of EAB presence. Random forest and ensemble model forecasts for the city of Edmonton, Alberta, Canada, indicated an area of potential concern for EAB. Our research strongly underscores the utility of comparative multi-model approaches in invasive risk assessments that could have important implications for pest surveillance and management.

## Introduction

Establishment and spread of non-native pests in evolutionarily naïve habitats are a cause of serious worldwide concern across societies from individuals to governments considering the potential ecological and economic damages that some non-native pests can inflict (Kenis et al. 2009, Gandhi and Herms 2010, Aukema et al. 2011, Nahrung and Carnegie 2020). The emerald ash borer (EAB), *Agrilus planipennis* Fairmaire (Coleoptera: Buprestidae), is a transcontinental wood-boring beetle species invasive in North America and eastern Europe (Cappaert et al. 2005, Valenta et al. 2017). Damage in North American urban and natural forests due to EAB may have been exceeded only by the chestnut blight disease that led to extirpation of chestnut, *Castanea dentata* (Marsh.) Borkh., in this region (Anagnostakis 1987). Originating in East Asia, EAB was first found in the US–Canada border cities of Detroit, Michigan, United States, and Windsor, Ontario, Canada, in 2002 (Cappaert et al. 2005). However, it is believed that EAB may have been present in this region at least a decade earlier (Siegert et al. 2007, 2014). Since its initial detection in Windsor, Ontario, EAB has spread far and wide including east to Bedford, Nova Scotia, west to Vancouver, British Columbia, and north to Avignon, Quebec (CFIA 2024). EAB is considered only a minor pest on East Asian ash species such as Manchurian ash (*Fraxinus mandschurica* Rupr.) (Wang et al. 2010), but in North America, all species of ash are considered susceptible, with some species more preferred than others (Anulewicz et al. 2007, 2008, Rebek et al. 2008, Pureswaran and Poland 2009, Herms and McCullough 2014). Specifically, extensive mortality of green ash (*F. pennsylvanica* Marshall), black ash (*F. nigra* Marshall), and white ash (*F. americana* L.) has been reported due to EAB infestations (Tanis and McCullough 2012, Burr and McCullough 2014, Herms and McCullough 2014, Klooster et al. 2014). Currently, EAB is found in 36 US states and 5 Canadian provinces (source: www.emeraldashborer.info). Studies estimate cumulative costs amounting to billions of dollars for removal and replacements of trees, insecticide applications, administering mitigation options, property devaluations, and timber market impacts due to EAB in North America (Kovacs et al. 2010, Aukema et al. 2011, McKenney et al. 2012). In Europe, EAB infestations have been found in European Russia (Moscow) in 2003, and in Ukraine in 2019, infesting planted non-native ash species of North American origin, specifically *F. pennsylvanica* (Drogvalenko et al. 2019). Native European ash (*F. excelsior* L.) in close proximity to *F. pennsylvanica* trees in Russia were also infested to a smaller extent (Orlova-Bienkowskaja 2014). An EAB invasion westward into the European Union, coinciding with the already extensive damage caused by ash dieback (*Hymenoscyphus fraxineus* (T.Kowalski) Baral, Queloz & Hosoya; ADB) (Enderle et al. 2019), or alternatively an invasion of ADB into North America, has the potential to cause a further decline in native ash populations (Davydenko et al. 2022).

EAB has a 1- or 2-yr life cycle depending on the location, oviposition timing, conspecific larval densities in the tree, tree health status (eg stress), and other factors (Cappaert et al. 2005, Wang et al. 2010). Gravid females lay eggs on the bark crevices of ash trees in the summer. Host selection for oviposition is influenced by tree condition, with ovipositing females preferring stressed trees (Tluczek et al. 2011, Jennings et al. 2014). The eggs hatch in 1 to 3 wk, then larvae bore through the bark and start feeding on the phloem and cambium (Wang et al. 2010, Poland et al. 2015). There are 4 larval instars in EAB. The last larval instar transforms into J-shape prepupa, which is typically the overwintering stage (Wang et al. 2010, Poland et al. 2015). The prepupa occurs in the first winter or the second winter of a 1- or 2-yr life cycle, respectively (Orlova-Bienkowskaja and Bieńkowski 2016). Extreme cold temperatures (<−30 °C) can be an important mortality factor of the prepupae as they are freeze-intolerant (Crosthwaite et al. 2011, Jones et al. 2017, Cuddington et al. 2018). However, recent research indicates that EAB prepupae display exceptional phenotypic plasticity that can help them survive winter temperatures of −50 °C (Duell et al. 2022). Pupation occurs in the spring, and eventually adults emerge in early to late summer through D-shaped exit holes, which are distinctive signs of an EAB infestation. Adult EAB have the capacity to fly long distances, some exceeding 20 km in flight-mill experiments (Taylor et al. 2010). However, the potential distances traveled could vary due to multiple factors such as host availability, landscape features, and EAB condition (eg age, sex, density, and mating status) (Cappaert et al. 2005, Taylor et al. 2010, Tussey et al. 2018). For instance, studies by Mercader et al. (2009) and Siegert et al. (2010) showed that new colonizations occurred at short distances (<~650 m) from brood trees. Tree damage occurs due to the serpentine galleries made by the larvae in the outer xylem, cambium, and phloem. This chewing activity interferes with sap and nutrient transport, effectively girdling the tree and resulting in tree death (Wang et al. 2010, Herms and McCullough 2014). Due to the cryptic nature of EAB biology, visual signs and symptoms of damage often appear 4 to 6 yr after the initial attack, limiting the possibility of an early intervention to help prevent further spread.

EAB has been extensively studied in the last 2 decades and several authors have elaborately described different facets of EAB ecology and spread (Crook and Mastro 2010, Wang et al. 2010, Bauer et al. 2014, Herms and McCullough 2014, Poland et al. 2015, Siegert et al. 2015, Valenta et al. 2017). A substantial amount of research work has addressed various aspects of EAB epidemiology in risk assessments including ash abundance (McCullough and Siegert 2014), spatial dynamics modeling (BenDor and Metcalf 2006, BenDor et al. 2006), short-distance and long-distance spread (Muirhead et al. 2006, Prasad et al. 2010, Iverson et al. 2016), climate-based risk assessments (Sobek-Swant et al. 2012b, DeSantis et al. 2013, Liang and Fei 2014, Cuddington et al. 2018), and time to invasion by EAB in the continental United States (Ward et al. 2020). Results of these studies suggest that various factors could contribute to the spread of EAB. For instance, proximity to neighboring infestations is a key indicator of invasion (Muirhead et al. 2006, Short et al. 2020, Ward et al. 2020). Spread of EAB can occur locally through active short-distance flights, and at long distances through passive human-assisted movement of EAB-infested materials such as firewood and nursery stock (Muirhead et al. 2006). However, rarely have the influences of both the local and long-distance infestations been considered in correlative spatial models for predicting EAB occurrences. Furthermore, scant consideration has been given in such models to combining multiple known or presumed influential drivers of EAB spread with biologically relevant climate variables, especially in Canada. Since Canada represents the northern expanding range of EAB in North America, it is possible that EAB–environment relationships may vary from the more southern, warmer portions of its invaded range. Teasing apart and quantifying the contributions of different factors influencing the distributions of invasive species such as EAB can provide new clues to pest spread dynamics. Additionally, given that spatial predictions will vary based on the model technique used, to our knowledge, none of the published modeling studies has considered comparing the predictive performances of multiple different models and their ensemble.

This study addresses the above knowledge gaps through the following 3 objectives, wherein the first 2 objectives focused on understanding response–predictor relationships (ie their strength and direction) and determining the best-fit combination of predictors and the third objective concerned predictive performances of multiple model types. Specifically, first we tested various predictors in logistic regression models including spatial, topographic/positional, transport pathways/human hotspots, host-related factors, and climate-related variables both individually and in combination. Second, we compared and interpreted the goodness-of-fit of the above suite of candidate logistic regression models to select the best candidate model and predictor set. Finally, we compared performances of different modeling techniques including logistic regression models, and an ensemble model, using the set of variables from the best candidate model. We illustrated the practical utility of our modeling efforts via predictions of potential EAB presences for the city of Edmonton, Alberta, Canada.

## Methods

### EAB Occurrence Data

Point occurrence data of EAB in Canada were collected and collated from the following unique sources:

1) EAB detection survey data from the Canadian Food Inspection Agency (CFIA; 2002 to 2018). In Canada, the CFIA has roles similar to the US Department of Agriculture’s Animal and Plant Health Inspection Service (USDA-APHIS) for plant protection. EAB detections by the CFIA were initially based on visual surveys (in the years following the first detection of EAB in Canada) but later a combination of methods including visual surveys, branch sampling, public reporting, and trapping was used. Since 2009, semiochemical-baited green prism traps have been the primary method utilized by the CFIA (Troy Kimoto, CFIA, personal communication). The majority of the data was from eastern Canada, with sparse data from central and western Canada.
2) Regional trap capture and/or branch sampling data from city forest managers for the cities of Ottawa, Ontario (2009, 2012 to 2017), Toronto, Ontario (2010 to 2011, 2016 to 2018), and Quebec City, Quebec (2017 to 2018).
3) EAB sampling data from 2 pest management companies, GDG Environment, Trois Rivières, Quebec (2016 to 2018) and BioForest, Sault Ste. Marie, Ontario (2010 to 2018). Data from GDG Environment were from trap captures and were mainly from the southern region of the province of Quebec including the Greater Montreal area, Granby, Sherbrooke, Trois Rivières, and Quebec City, and from 2 parks in Toronto, Ontario (2018). Sampling data from BioForest included information on adult trap captures (2011 to 2018) and branch sampling results for larvae (2010 to 2013) from the town of Oakville in the Greater Toronto Area in Ontario.

Visual surveys typically involved finding trees showing characteristic indicators of EAB infestations such as D-shaped exit holes, crown dieback/decline, serpentine larval galleries, and epicormic shoots. Adults were captured using sticky prism traps or funnel traps baited with the host volatile, (3Z)-hexenol, and/or the female-produced pheromone, (3Z)-lactone (Ryall 2015, Bullas-Appleton et al. 2019). Branch sampling protocol was based on Ryall et al. (2011). Surveillance objectives (eg pre-detection surveys in areas where EAB was not confirmed, post-detection delimitation surveys after EAB was detected, trapping to guide pest management actions), survey approaches employed, spatiotemporal sampling intensity, and data reporting formats differed from one source to the other, but 1 or more measures of both positive detections (eg EAB survey result recorded as “detected,” number of adults caught, presence or number of larval galleries) and negative detections (eg EAB survey result recorded as “not detected,” zero adult counts, absence of larval galleries) were recorded.

**Fig. 1.**
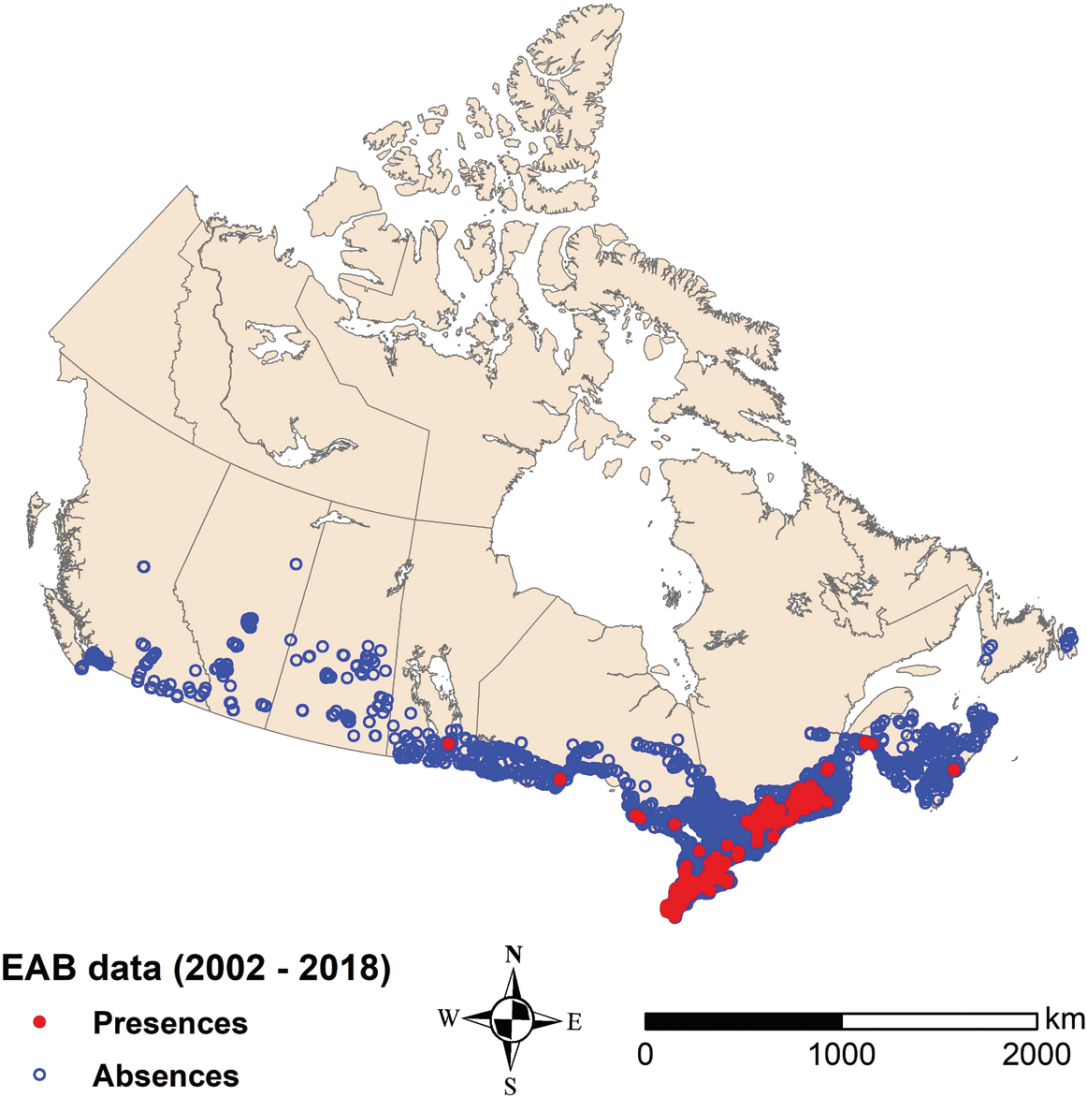
EAB occurrence hexagons (presence, absence) from 2002 to 2018 in Canada used in modeling (*n* = 21,984).

Considerable effort was put toward collecting EAB occurrence information. However, our data set does not represent a complete record of EAB spatiotemporal occurrence in eastern Canada either because the data were nonexistent due to survey limitations (eg imperfect detection), or because it was not possible to obtain empirical EAB occurrence records from all existing data sources, resulting in some data gaps. Nevertheless, we believe that the overall spatiotemporal data coverage (2002 to 2018) is sufficiently extensive as it includes all major infestation epicenters prior to 2019 (Fig. 1).

Using QGIS v. 3.4 and ESRI ArcMap 10.5.1 we created a 1-km^2^ hexagonal tessellation (hereafter, hexagon) over the full extent of Canada and overlaid the EAB occurrence data to derive a simplified binary coded response variable indicating presence or absence of EAB in each hexagon, for any given year. Here, presences indicate that adults and/or immatures were found in a hexagon, whereas absences indicate that no such records were found. A total of 21,984 observations (presence hexagons = 1,519; absence hexagons = 20,465) from 2002 to 2018 were used in the analyses (Fig. 1).

### Developing Predictor Variables

We classified the predictors into 5 categories or drivers namely those representing infestation pressure, location/topography, transport corridors/potential hotspots (ie domestic invasion pathways), host-related factors, and weather-based parameters (Table 1).

**Table 1.** Predictor variables considered in developing models of EAB occurrences

Predictor variable (*short name*; unit)^a^	Description and reference(s)
**Infestation pressure**	
Spatial term (*spatial;* dimensionless)	A negative exponential dispersal kernel adapted from the “force of invasion” concept of Havel et al. (Havel et al. 2002). A negative exponential form of the kernel was modified by a parameter “*a*” that changed the shape of the dispersal curve (Eq. (1); Supplementary Fig. S1). Infestation pressure in the neighborhood is an important driver of EAB spread (Muirhead et al. 2006, Prasad et al. 2010, Short et al. 2020, Ward et al. 2020).
**Position/topography**	
Latitude of the current year infestation (*lon*; decimal degrees)	Latitude of the centroid of the hexagon.
Longitude of the current year infestation (*lon*; decimal degrees)	Longitude of the centroid of the hexagon.
Mean elevation (*Elev*; m)	Topographic characteristics influence the microclimate, host quality, host composition and abundance, connectivity, and moisture relations affecting EAB population growth and spread (Crocker et al. 2005, 2010).
Mean slope (*slope*; degrees)	
Mean aspect (*aspect*; −1 to 1, south to north)	
**Transport corridors/potential hotspots**	
Road length (*Roads(log10)*; m)	Roads and railway networks contribute to long-distance spread of EAB via transport of infested materials (Prasad et al. 2010, Yemshanov et al. 2012, Short et al. 2020). High human population densities and presence of recreational areas such as campgrounds and parks are associated with a higher risk of EAB occurrence (Crocker et al. 2011, Ward et al. 2020).
Railway track length (*Rail (log10)*; m)	
# Rail stations (*Stations*; dimensionless)	
Number of campgrounds per hexagon (*Campgrounds*; dimensionless).	
Percent area covered by national or provincial parks (*Parks*; dimensionless).	
Human population density (*Popdensity(log10)*; dimensionless)	
**Host-related factors**	
Mean percent crown closure (*Crown*; dimensionless)	Adult beetles prefer open and sunny conditions as ash trees in such areas are frequently attacked (Wang et al. 2010). Volumes of ash and non-ash trees are associated with time to invasion by EAB (Ward et al. 2020). Here we use broad-leaf biomass (%) as a proxy for biomass of ash. Urban areas and forests along highways may be at a higher risk of EAB-associated damage due to higher potential for human-mediated spread of EAB via infested materials and beetles hitchhiking vehicles (Prasad et al. 2010).
Percent vegetated and treed *(Treed*; dimensionless)	
Mean percent of biomass represented by broad-leaf species (*Broad-leaf*; dimensionless)	
Landcover^b^ (*Landcover class*; dichotomous)	
**Weather-based parameters**	
Accumulated degree-days >= 10 °C (*deg_days*; degree-days)	Development rate is influenced by temperature (BenDor et al. 2006). Warmer summer conditions increase adult emergence and flight activity of EAB (Wang et al. 2010). Seasonal activity of EAB is associated with accumulated degree-days (Brown-Rytlewski and Wilson 2004, Poland et al. 2011). Sustained extreme cold conditions (<−30 °C) could limit EAB distributions (Crosthwaite et al. 2011, DeSantis et al. 2013, Jones et al. 2017).
Coldest quarter mean temperature (*cqmt*; °C)	

^a^A list of data sources used in this analysis is presented in the supplementary information section (Supplementary Table S1).

^b^Based on Annual Land Cover Time Series of Canada for 2000 to 2011 (25 classes). Dichotomous, 1 = “Urban and built-up” or “mixed needle-leaved cold deciduous forest, medium density”; 0 = other classes.

Regarding infestation pressure, invasive forest insects such as EAB exhibit stratified dispersal in invaded habitats, meaning short-distance active flights that result in infestations locally, and long-distance human-assisted movements that result in spatially disparate populations far from the original infestation epicenter (Herms and McCullough 2014). Local dispersal usually results in new EAB infestations within a kilometer of the original infested tree (Mercader et al. 2009, Siegert et al. 2010). New infestations have also been found hundreds of kilometers from the nearest epicenter, likely facilitated by humans (Ward et al. 2020).

To account for spatiotemporal autocorrelation stemming from short- and long-distance dispersal of EAB, we considered lagged spatiotemporal neighborhoods of EAB presences via a dispersal kernel and used this variable as a predictor in various models. We made 3 assumptions while developing the dispersal kernel. First, we assumed that since EAB has a 1- or a 2-yr life cycle depending on various factors, presence of infestations in the neighborhood (source) prior to the current year would initiate a new colonization event in another hexagon (sink). The beginning colonization event in the previous year(s) in the sink hexagon would then result in detections of EAB (visual signs of tree infestations, larval galleries, and/or adult trap captures) in the current year in that hexagon. Second, we assumed that the influence of the current year’s infestations and their attribution to fresh detections of incipient infestations (ie larvae) in neighboring hexagons the same year could be uncertain and minimal given the influence of colder climates in Canada on EAB development. So, we did not consider the current year’s spatial neighborhood in the development of the dispersal kernel. Although an untreated EAB-positive hexagon can be a potential long-term source of outgoing beetles, to minimize computational complexity, we restricted our spatial neighborhood structure to 2 previous years. And the third assumption that we made was that the EAB trap captures in a hexagon indicate tree infestations in that hexagon. Attractive radius of EAB attractant-baited traps is just a few tens of meters (Wittman et al. 2021), so infested trees would likely be in the vicinity of the traps. It is possible that some of the adults trapped were transient individuals from geographically distant areas; however, their numbers in our trap capture data are unknown.

We adapted the “force of invasion” concept to model the dispersal kernel of EAB (Havel et al. 2002, Meentemeyer et al. 2008), represented as the following spatial variable:


$$\begin{array}{l} Spatial=\sum_{k=1}^{N}exp\left(\frac{-d_{ik}}{a}\right) \end{array}$$ (1)

Where *d*_*ik*_ is the standardized geodetic distance (0 – 1) between the centroid of current hexagon *i* (infested or not) in the year *t* and the centroid of an infested hexagon *k* in the year (*t* – 1) or in the year (*t* – 2) taking into account 1- or 2-yr life cycle of EAB; *N* represents all the infested hexagons in year (*t* – 1) and year (*t* – 2) within a distance of 300 km, which was chosen subjectively to take into account passive, anthropogenic dispersal of EAB that can be hundreds of kilometers at random from an epicenter (Muirhead et al. 2006, Ward et al. 2020); term *a* represents a correction modifying the shape of the dispersal kernel (see Supplementary Fig. S1). Infestations nearer to the target hexagon weigh more than farther infestations in terms of colonization potential when *a* is lower (Supplementary Fig. S1). On the contrary, when *a* values are higher, there is a comparatively greater probability of invasion from farther infestations (Supplementary Fig. S1; Havel et al. 2002). Although any number of *a* values could have been tested, for computational simplicity we used 4 different values in our analyses: 1, 3, 5, and 10. The spatial variable was created in SAS v. 9.3 (SAS Institute, Cary, North Carolina).

A detailed description of the other 4 categories (ie driver groups) of predictor variables used in the development of models is given in Table 1. Links to initial data sources can be found in the supplementary information section (Supplementary Table S1). To facilitate data modeling, multiple variables were extracted, compiled, and characterized across a 1-km^2^ hexagonal grid using different methodologies in ArcGIS, unless otherwise noted. All ArcGIS operations were performed in Canada Lambert Conformal Conic (NAD83) projection.

For each hexagon, mean values were extracted from raster layers representing topographic features (elevation, slope, and aspect) and host-related vegetation cover variables (Beaudoin et al. 2017), which were originally aggregated at a common resolution of 250 m. To determine the land cover category that best characterized each hexagon, we used zonal statistical to calculate the majority class for each hexagon based on the 250 m annual land cover time series of Canada (2000 to 2011), which is comprised of 25 classes (Pouliot et al. 2014) (Supplementary Table S1). We then used R v. 4.2.3 (R Core Team 2023) to binary code the land cover classification for each hexagon as either 1 (“urban and built-up” or “mixed needle-leaved cold deciduous forest, medium density”) or 0 (all other classes). Initial insights gained from examining the descriptive statistics indicated that this classification closely reflected the distributional pattern of our EAB presence–absence data. Maximum road length (m) per hexagon was determined by tabulating the intersection of the hexagon layer with road network data from both Statistics Canada and the National Road Network (see Supplementary Table S1), then selecting the maximum value for road length from either source. Railway track length (m) and count of National Railway Network (NRWN) stations per hexagon were each determined by calculating the intersection of the relevant NRWN data (Supplementary Table S1) and the hexagon layer. Human population density per hexagon was determined by downloading raster data from the European Commission’s Joint Research Centre (2015) and calculating population count within the hexagon. Road length, railway track length, and human population density were log-transformed (log_10_ + 1) prior to running the analyses to account for zero values and outliers. Campground data were compiled from ArcGIS Online, via Google Map listings, and provincial tourism websites, among other sources. These data were intersected with the hexagon layer to determine the count of campgrounds per hexagon. National parks data were downloaded from Parks Canada and provincial parks/protected areas data were collected from provincial websites (Supplementary Table S1). These data sets were combined and dissolved to create a complete parks layer, then the total area occupied by parks per hexagon was determined by tabulating the intersection of the parks layer with the hexagon layer. Weather variables were generated using BioSIM v. 11 (Supplementary Table S1).

### Logistic Regression Models Using Single Versus Multiple Predictors Across Driver Groups

We first developed different logistic regression models using (a) “single” predictors (hereafter referred to as “single predictor” or “individual” logistic regression models) and (b) a combination of predictors belonging to the same predictor category (driver group), after accounting for spatiotemporal dependence by including the best-fit spatial term in the models (Eqs. (2) and (3)). We chose logistic regression to assess relationships between EAB occurrences and the different predictors because it is a widely used statistical method with well-developed theory and easily interpreted parameters (Agresti 2013). Our final model was created by combining predictors across different categories to develop a multivariable logistic model, as described by Eq. (3) and the probability of EAB presence was assessed through Eq. (4):


$$\begin{array}{l} L_{i}=log\left(\frac{prob_{i}}{1-\;prob_{i}}\right)=\;\beta_{0}+\beta_{1}\sum_{k=1}^{N}\exp\left(\frac{-d_{ik}}{a}\right)+\;\beta_{2}x_{2} \end{array}$$ (2)

$$\begin{array}{l} L_{i}=log\left(\frac{prob_{i}}{1-\;prob_{i}}\right)=\;\beta_{0}+\beta_{1}\sum_{k=1}^{N}\exp\left(\frac{-d_{ik}}{a}\right)+\sum_{j=2}^{\;p}\beta_{j}x_{j} \end{array}$$ (3)

$$\begin{array}{l} prob_{i}=\;\frac{\exp(L_{i})}{1+exp(L_{i})}\; \end{array}$$ (4)

Here, *L*_*i*_ is the log odds (logit), *prob*_*i*_ is the probability of EAB presence in hexagon *i* given the predictors, β_0_ is the intercept, β_1_ is the parameter estimate for the spatial term $\sum_{k=1}^{N}\exp\left(\frac{-d_{ik}}{a}\right)$ (see Eq. (1) for the description of the terms), β_2_ is the parameter estimate of the predictor (*x*_2_) in a single predictor model (Eq. (2)), β_*j*_ is the parameter estimate of the *j*th predictor (*x*_*j*_) in a multivariable model (Eq. (3)), and *p* represents the number of predictors used. For the spatial term, we chose the value of *a* among 1, 3, 5, and 10 that resulted in the smallest Akaike Information Criterion (AIC) value in individual logistic regressions (Akaike 1974). A smaller AIC value indicates a better model fit to data. To avoid potential issues due to sample imbalance between presences and absences, we randomly sampled an equal number of absences (*n* = 1,325), which reflected the spatial distribution of the overall data (ie not skewed geographically), as the presences (*n* = 1,325) from across all years (ie 2004 to 2018). Presence/absence data from 2002 and 2003 were only used to characterize lagged spatial neighborhoods for the year 2004; they were not used as response data in models because EAB occurrence records do not exist prior to 2002, so lagged spatial neighborhoods could not be constructed for those years. Logistic models were run in R v. 4.2.3 using the *glm* function with *logit* link. The predictor set used in a multivariable model for a given driver group was selected by first assessing pairwise Pearson correlation coefficients (*r* < |0.75|) for continuous predictors, then checking for multicollinearity using variance inflation factor (VIF) scores (<5). When 2 variables were collinear (which occurred only for continuous host-related variables), AIC values of competing models were used to retain the best predictor. We performed variable selection through backward elimination strategy using the *stepAIC* function in *MASS* package in R (Venables and Ripley 2002). Goodness-of-fit of the logistic model predictions relative to sample data was assessed using AIC values. AIC values from candidate multivariable logistic models for different driver groups were compared with the AIC value from the final multivariable model to determine the best model among the set using the *AICcmodavg* R package (Mazerolle 2023).

### Using Different Model Types for Predicting EAB Occurrences

Testing multiple model types is relevant when the highest possible predictive accuracy is the desired goal (Sharma and Jackson 2008, Hao et al. 2020). Using a combination of well-established parametric and nonparametric models of different types (statistical, machine learning, etc.) may yield a better performance than any individual model type (Araújo and New 2007). Therefore, we wanted to determine the best-performing individual model among a set of model types, then assess whether an ensemble of best-performing individual models would provide a better predictive performance than any individual model alone. We used the *Biomod2* (v. 4.2-2) package in R to develop and compare the predictive accuracies of various models described below with an ensemble model (Thuiller et al. 2024). The different model types that we considered for EAB presence–absence predictions are correlative models that come under the species distribution modeling (SDM) framework for predicting species distributions. They include generalized linear models (logistic regression models; GLM), multivariate adaptive regression splines (MARS), artificial neural networks (ANN), classification tree analysis (CTA), generalized boosting models (GBM), and random forest (RF). While GLM and MARS are regression-based, the latter method uses smoothing splines and can model complex relationships between the response variable and the predictors (McCullagh and Nelder 1989, Hastie and Tibshirani 1990, Friedman 1991). ANN is a machine learning method that flexibly “learns” complex relationships between predictors and the response variable by adjusting weights and biases in the interconnected neurons (Ripley 2007). Thus, the error between the response and the prediction is progressively reduced. CTA, GBM, and RF are tree-based algorithms (Breiman et al. 1984, Breiman 2001, Elith et al. 2008). More specifically, although both GBM and RF are ensemble modeling methods that use random bootstrap samples, final fitted values in GBM are derived by summing contributions of individual decision trees in a “forward, stagewise” manner through consecutive modeling of residuals from the previous tree iteratively and via recalculation of fitted values (Elith et al. 2008). RF combines predictions from multiple decision trees (eg majority voting in the case of binary responses) that in turn use a random set of predictors from the predictor set at each node as the tree is grown (Breiman 2001). Parameter settings used for modeling are included in the supplementary information section (Supplementary Appendix S1) and description of parameters can be found in the documentation of *biomod2* (Thuiller et al. 2024). We used True Skill Statistic (TSS; true positive rate + true negative rate – 1) scores to assess the performance of models (Allouche et al. 2006, Sambaraju and Côté 2021). These scores indicate the ability of a model to correctly classify presences and absences. The values of TSS range from −1 (worst) to 1 (best), and models that show higher values of TSS (>0.6) were considered good models (Préau et al. 2019).

To minimize potential uncertainties and biases associated with variations in sampling intensity and spatiotemporal gaps in sampling, we first randomized the presence–absence data (2004 to 2018) and split EAB presence data into 2 parts, 80% (*n* = 1,060 presences) for training purposes and 20% (*n* = 265 presences) for evaluation purposes. Absence data were similarly divided into 2 portions, 80% (*n* = 14,611 absences) for training and 20% (*n* = 3,653) for evaluation. We sampled 10 sets of absences (*n* = 1,060 per set) and joined them to the training presence data (*N*_training_ = 2,120 presence–absence observations per set). From the evaluation absence set, 265 absences were sampled and joined to the evaluation presence data (*N*_evaluation_ = 530). The different modeling methods were run on the training data with 70% and 30% random splits of the data for calibration and internal cross-validation of models, respectively. A total of 180 models were developed [6 models × 10 repetitions (each with a different absence set) × 3 cross-validation runs]. The number of iterations per model used is consistent with previous studies (Gallien et al. 2012, Préau et al. 2019, Ramírez-Preciado et al. 2019, Sharma et al. 2021). We developed an ensemble model from individual models whose TSS scores were >0.6 (*n* = 78). Performances of individual and ensemble models were tested on the evaluation data. For ensemble models, simple voting or *committee averaging* method was used to determine the predicted response (Thuiller et al. 2024). In this method, individual predictions (1s and 0s of EAB occurrences) of selected best-performing/reliable models (TSS > 0.6) are used to produce consensus binary predictions based on majority agreement among individual model predictions (Gallien et al. 2012). Joint tests of pairwise means and comparison of least square mean TSS scores for validation and evaluation sets were separately performed using *emmeans* and *multcomp* packages in R (Hothorn et al. 2021, Lenth et al. 2021).

Variable importance (VI) scores, which indicate influence of individual predictors on the model (Thuiller et al. 2024), were computed for all the models. In *biomod2*, VIs are calculated based on a permutation-based method. In this approach, values of a given predictor are shuffled in the training data set keeping the values of other predictors unchanged, and model performances are evaluated with the newly shuffled data. Then, the original and shuffled model predictions are assessed through Pearson’s correlation and a VI score is returned as 1 – corr. Thus, the greater the difference in model predictions (the lower the correlation coefficient and the higher the value of the VI score) the greater the influence of the predictor on the model performance (or vice versa) (Thuiller et al. 2024). We also plotted the mean probabilities of EAB presence, averaged across all cross-validation runs and absence sets per individual model type, at different values of select influential predictors determined based on VI analyses. Plotting did not include ensemble models because committee averaging method in *biomod2* ensemble modeling does not provide predictions on a raw probability scale (Thuiller et al. 2024).

Finally, a practical application of our modeling effort is demonstrated via forecasts of the likelihood of EAB occurrence for the city of Edmonton, Alberta, Canada. Edmonton was chosen because it is a large urban center with more than 90,000 ash trees (https://data.edmonton.ca/Environmental-Services/Trees/eecg-fc54/about_data) at risk of EAB infestations. Therefore, ecological and economic impacts can be very significant (Hope et al. 2021). As a first step, hexagons intersecting the city limits of Edmonton (https://data.edmonton.ca/Administrative/City-of-Edmonton-Corporate-Boundary-current-/qqvh-dp5m/about_data) were retained for analysis. Then using the RF and ensemble models (*n* = 30 each), we predicted EAB presence for each hexagon via the *BIOMOD_Projection* function and *BIOMOD_EnsembleForecasting* function (for ensemble models only) (Thuiller et al. 2020). Since no confirmed EAB detections were recorded within a 300-km radius of Edmonton as of 2023, we set the value of the *Spatial term* to zero for modeling purposes. We extracted the centroids of the hexagons and calculated the accumulated degree-days (>10 °C) using 2023 weather data from BioSIM v. 11. Rest of the variables from the best predictor set were generated as previously described in the “Developing Predictor Variables” section.

## Results

### Logistic Regression Models Using Single Versus Multiple Predictors Across Driver Groups

We first present the results of single predictor logistic regression models that were intended to better understand the strength and direction of the relationship between individual predictors, after accounting for spatiotemporal dependencies in response data via the best-fit spatial term, and the probability of EAB presence. Then, we examine a combination of predictors within a given driver category. Finally, we report results of a multivariable model that included predictors from different driver categories.

Details of single predictor logistic regression models are presented in Table 2 and described here. Presence of infestations in the neighborhood in previous years (infestation pressure), quantified by the *Spatial* variable (Eq. (1)), was positively associated with EAB infestations in the current year. However, giving a proportionately greater weight to farther infestations (ie *a* = 3, 5, or 10) compared to the unweighted dispersal kernel (*a* = 1) worsened the fit of the models (AIC values: 3,507.9 to 3,532.3 vs. 3,437.6; Table 2*Spatial* portion). Therefore, all single and multivariable models included the best-fit spatial term (*a* = 1). With regard to position/topography, latitude did not influence the probability of EAB presence. Infestations occurred more frequently in eastern sampling sites than in western sampling sites between 2004 and 2018 in Canada. Presences of EAB were negatively associated with increasing elevations. Hexagons with higher slopes and south-facing aspects had a greater probability of an EAB infestation. For the transport group, greater road or rail track length, and higher number of rail stations per hexagon were all associated with a higher probability of EAB presence. However, human activity indicators were not always associated with EAB occurrence. For instance, while high human population density increased the probability of EAB presence, percent area of parks per hexagon was unrelated to EAB presence. Campground occurrences were negatively associated with the presence of an EAB infestation (Table 2). For the host-related variables, we found that mean percent crown closure, mean percent vegetated and treed, and mean percent of biomass represented by broad-leaf species did not influence the likelihood of an EAB infestation after accounting for past spatiotemporal infestations. EAB infestations were highly associated with landcover class wherein urban areas and medium-density mixed forests were linked to a higher likelihood of EAB presence. Finally for the weather variables, the higher the cumulative accumulation of degree-days (>10 °C) during a given year, the greater the odds that a hexagon would contain an EAB infestation. The probability of EAB presence increased with warmer winter temperatures (Table 2).

**Table 2.** Logistic regression models (Eq. (2)) associating different predictors, individually or in a group, with EAB infestations (2004 to 2018) in Canada

Predictor variable(s)	Estimate ± SE	Odds ratio (95% CI)^a^	*Z*	*P*	AIC^b^
Intercept-only	0.000 ± 0.039	—	0.00	1	3,675.7
Infestation pressure					
*Spatial*					
** *a* = 1**	**0.009 ± 0.001**	**1.009 (1.008 to 1.010)**	**14.73**	**<0.001**	**3,437.6**
*a* = 3	0.007 ± 0.001	1.007 (1.006 to 1.008)	12.60	<0.001	3,507.9
*a* = 5	0.006 ± 0.001	1.006 (1.005 to 1.007)	12.11	<0.001	3,521.9
*a* = 10	0.006 ± 0.001	1.006 (1.005 to 1.007)	11.73	<0.001	3,532.3
Position/topography^c^					
*Lat*	−*0.041 ± 0.029*	*0.960 (0.906 to 1.015)*	−1.42	*0.156*	*3,437.5*
*Long*	0.092 ± 0.010	1.096 (1.075 to 1.119)	8.89	<0.001	3,318.4
***Elev***	**−0.011 ± 0.001**	**0.990 (0.988** to **0.991)**	**−16.71**	**<0.001**	**3,052.8**
*Slope*	0.102 ± 0.041	1.107 (1.023 to 1.199)	2.51	0.012	3,433.3
*Aspect*	−0.600 ± 0.099	0.549 (0.452 to 0.666)	−6.06	<0.001	3,402.2
***Combined—topography*** ^***d,e***^	—	—	—	—	**2,998.8**
Transport corridors/potential hotspots^c^					
*Roads(log10)*	0.612 ± 0.059	1.845 (1.647 to 2.079)	10.31	<0.001	3,302.7
*Rail(log10)*	0.142 ± 0.034	1.152 (1.078 to 1.232)	4.17	<0.001	3,422.0
*Stations*	0.975 ± 0.263	2.650 (1.614 to 4.555)	3.70	<0.001	3,423.7
*Campgrounds*	−1.042 ± 0.277	0.353 (0.199 to 0.593)	−3.76	<0.001	3,422.9
*Parks*	−*0.008 ± 0.005*	*0.992 (0.983* to *1.001)*	−1.76	*0.079*	*3,436.2*
***Popdensity (log10)***	**0.659 ± 0.036**	**1.932 (1.801 to 2.076)**	**18.17**	**<0.001**	**3,051.4**
***Combined—Transport*** ^***d,e***^	—	—	—	—	**3,044.3**
Host-related factors^c^					
*Crown*	*0.001 ± 0.003*	*1.001 (0.996* to *1.006)*	0.29	*0.774*	*3,439.5*
*Treed*	−*0.002 ± 0.002*	*0.998 (0.995 to 1.002)*	−0.98	*0.329*	*3,438.6*
*Broad-leaf*	−*0.001 ± 0.002*	*0.999 (0.995 to 1.003)*	−0.48	*0.631*	*3,439.3*
***Landcover class***	**1.860 ± 0.097**	**6.423 (5.320 to 7.783)**	**19.16**	**<0.001**	**3,016.1**
*Combined—Host* ^***d,e***^					**2,943.2**
Weather-based parameters^c^					
***Deg_days***	**0.004 ± 0.000**	**1.004 (1.004** to **1.005)**	**16.32**	**<0.001**	**3,114.7**
*CQMT*	0.060 ± 0.014	1.062 (1.034 to 1.090)	4.46	<0.001	3,419.3
***Combined—weather*** ^***d,e***^					**3,109.8**
*All variables combined* ^f^					**2,481.7**

^a^Odds ratio indicates the change in odds of finding EAB as a function of a unit change in a given predictor value. An odds ratio value >1 (or <1) indicates higher (or lower) odds of EAB presence.

^b^A lower AIC value indicates a better fit.

^c^Spatiotemporal dependence term (*Spatial*, *a* = 1) was included in single predictor and multivariable models. A “single predictor” variable in bold indicates the best predictor, after accounting for spatiotemporal dependence, among the set of single predictors within a given driver group. A multivariable model (a group of predictors combined) per driver group in bold indicates that that model is a better fit to response data than any of the single predictor models.

^d^“*Combined—topography*” model included *Spatial* (*a* = 1), *Elev*, *Slope*, and *Aspect*; “*Combined*—*Transport*” model included *Spatial* (*a* = 1), *Campgrounds*, and *Popdensity (log10)*; “*Combined*—*Host*” model included *Spatial* (*a* = 1), *Crown*, and *landcover class*; and “*Combined*—*weather*” model included *Deg_days* and *CQMT*.

^e^Predictors in multivariable models were considered after considering multicollinearity (|*r*| > 0.75), VIF scores (<5), and removal of nonsignificant terms.

^f^Only *Spatial*, *Elev*, *Slope*, *Campgrounds*, *Popdensity(log10)*, *Crown*, *Landcover class*, and *Deg_days* were included.

Without exception, after consideration of multicollinearity (|*r*| < 0.75), VIF scores (<5), and removal of nonsignificant terms (*P *> 0.05), we found that combining relevant terms of a given set of variables within a particular driver group provided a better fit than terms used singly, after considering spatiotemporal dependencies, in individual logistic regression models (Table 2). The best candidate model, selected through the backward elimination procedure on predictors belonging to different driver groups, and meeting the variable selection criteria, consisted of 8 predictors (Tables 3 and 4). This model had the lowest AIC value of 2,481.7 compared with other models that consisted of best predictors belonging to a given driver group (Tables 2–4).

**Table 3.** Selection of the best model among a set of candidate models to predict EAB occurrences^a^^,^^b^

Models	*K*	AIC	∆AIC	AICWt	LL
All variables combined	**9**	**2,481.7**	**0.0**	**1.00**	**−1,231.8**
Combined—host	4	2,943.2	461.5	0.00	−1,467.6
Combined—topography	5	2,998.2	517.2	0.00	−1,494.4
Combined—transport	4	3,044.3	562.6	0.00	−1,518.2
Combined—weather	3	3,109.8	628.1	0.00	−1,551.9
Spatial term	2	3,437.6	955.9	0.00	−1,716.8
Intercept-only	1	3,675.7	1,194.0	0.00	−1,836.8

^a^“*K*” represents the number of predictors in a given model.

^b^A lower AIC value indicates a better fit. A difference in AIC values (∆AIC) of >2 between any 2 candidate models indicates that the model with lower AIC value has greater support. AICWt = Akaike weight (value of 1 indicates that the candidate model is the best); LL = log-likelihood.

**Table 4.** Best multivariable logistic regression model (see Table 3) associating different predictors with presence/absence of EAB infestations (2004 to 2018) in Canada

Term^a^^,^^b^	Estimate	Odd ratio^c^	*Z*	*P*	95% CI
*Intercept*	−6.116 ± 0.467	—	−13.09	<0.001	[−7.042, −5.210]
*Spatial*	0.009 ± 0.001	1.009 (1.007 to 1.011)	7.33	<0.001	[0.007, 0.011]
*Elev*	−0.008 ± 0.001	0.992 (0.991 to 0.994)	−10.59	<0.001	[−0.009, −0.006]
*Slope*	0.154 ± 0.058	1.166 (1.040 to 1.307)	2.63	0.009	[0.039, 0.268]
*Campgrounds*	−0.803 ± 0.304	0.448 (0.241 to 0.797)	−2.64	0.008	[−1.425, −0.227]
*Popdensity(log10)*	0.403 ± 0.060	1.496 (1.332 to 1.682)	6.76	<0.001	[0.286, 0.520]
*Crown*	0.046 ± 0.004	1.047 (1.039 to 1.055)	11.82	<0.001	[0.038, 0.053]
*Landcover class*	1.147 ± 0.158	3.149 (2.315 to 4.296)	7.27	<0.001	[0.839, 1.458]
*Deg_days*	0.004 ± 0.000	1.004 (1.003 to 1.004)	10.85	<0.001	[0.003, 0.004]

^a^AIC value of the model = 2,481.7.

^b^Variables considered after accounting for multicollinearity (|*r*| > 0.75) and VIF scores (<5).

^c^Odds ratio indicates the change in odds of finding EAB as a function of a unit change in a given predictor value. An odds ratio value >1 (or <1) indicates higher (or lower) odds of EAB presence.

The best candidate logistic regression model included spatial, topographic (slope, elevation), transport/hotspot (campgrounds and human population density), host-related (mean crown closure, landcover), and weather-based (degree-days) variables (Table 4). The direction of the relationship of all the variables, except mean crown closure, with the presence of EAB in the final model (Table 4) remained consistent with the single-variable model (Table 2). After adjusting for other predictors, mean crown closure showed a positive association with the probability of EAB presence that was not observed in the corresponding single predictor model.

### Using Different Model Types for Predicting EAB Occurrences

**Fig. 2.**
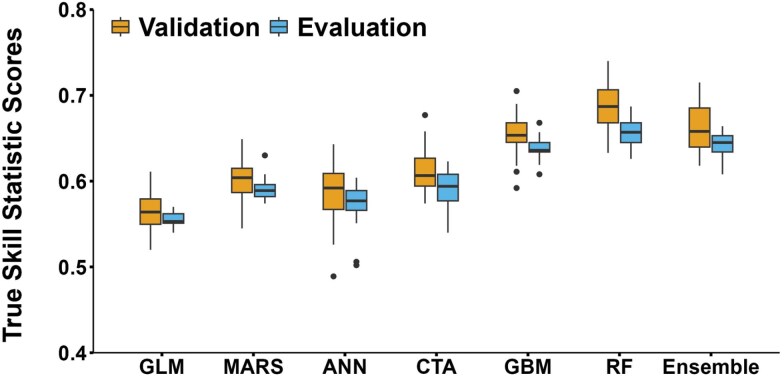
Comparative distributions of TSS scores of different modeling techniques on validation and evaluation sets for predicting the probability of presence of the EAB (*n* = 30, except ANN where *n* = 29). Each box represents mid 50% of the TSS data (interquartile range, IQR) for a given model and the horizontal bar within the box indicates the median TSS for a given model. The whiskers extend to minimum and maximum TSS values within 1.5 times beyond the box edges and the dots represent potential outliers. GLM = generalized linear model; MARS = multiple adaptive regression splines; ANN = artificial neural networks; CTA = classification tree analysis; GBM = generalized boosting model; RF = random forest. The TSS values range from -1 (worst) to 1 (best).

**Fig. 3.**
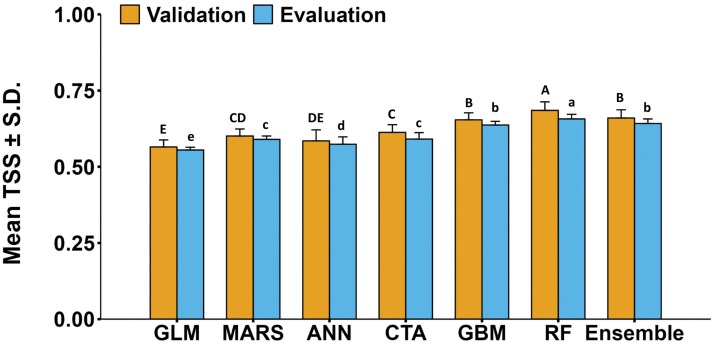
Comparison of mean TSS values for different modeling techniques to predict EAB presences. GLM = generalized linear model; MARS = multiple adaptive regression splines; ANN = artificial neural networks; CTA = classification tree analysis; GBM = generalized boosting model; RF = random forest. The TSS values range from -1 (worst) to 1 (best).

Distribution of TSS values for different model types across 10 presence–absence sets times 3 cross-validation runs per presence–absence set (*N* = 30 runs per model; see “Methods” for details) for validation and evaluation data is shown in Fig. 2. For ANN, of the 30 model runs, 1 failed, so the TSS values are from 29 models. There were significant differences in the mean TSS values by model type for validation (*F* = 80.2, df = 6, 202, *P *< 0.001) and evaluation (*F* = 174.2, df = 6, 202, *P < *0.001) data sets (Fig. 3). Comparison of TSS distributions across the 7 investigated model types (GLM, MARS, ANN, CTA, GBM, RF, and the ensemble model) showed that the RF algorithm performed better than the other models including the ensemble model (Figs. 2 and 3). Average TSS values (±SD) for the RF algorithm were 0.69 ± 0.03 (validation) and 0.66 ± 0.02 (evaluation), whereas the ensemble model was marginally less precise than the RF model with average TSS values of 0.66 ± 0.03 (validation) and 0.64 ± 0.02 (evaluation) (Fig. 3). CTA and GBM performed less well than RF but had higher average TSS scores than GLM and ANN models (Fig. 3). MARS performed significantly poorer than GBM, RF, and ensemble.

**Fig. 4.**
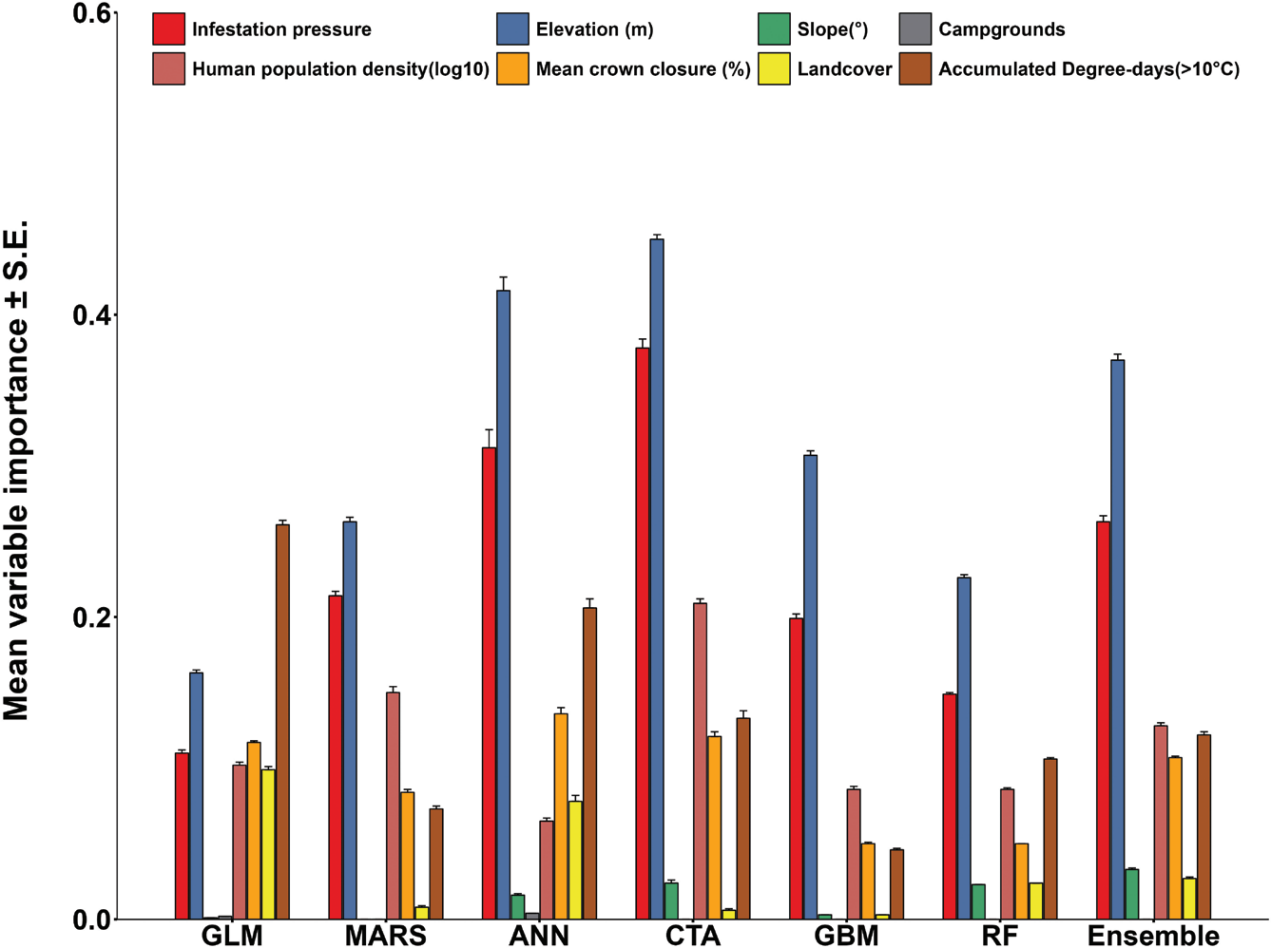
Variable importance scores of different variables indicating their influence on model predictions (*n*_runs_ = 6). GLM = generalized linear model; MARS = multiple adaptive regression splines; ANN = artificial neural networks; CTA = classification tree analysis; GBM = generalized boosting model; RF = random forest.

VI scores differed among the model types (Fig. 4). However, elevation [VI range: 0.16 to 0.45] and spatial term (infestation pressure; [0.11 to 0.38]) were consistently important and informative predictors of EAB presences across all 7 model types including the ensemble model. Human population density (log10) [0.07 to 0.21], accumulated degree-days (>10 °C) [0.05 to 0.26], and mean crown closure [0.05 to 0.14] were also influential variables and their contribution to predicting EAB presences was strong. Landcover was not a notable contributor to model predictions in the case of MARS, CTA, and GBM [VI < 0.02]. Slope was generally of low importance overall [VI < 0.03], while the campgrounds variable was the least important among the set of predictors used in this analysis [VI < 0.004].

**Fig. 5.**
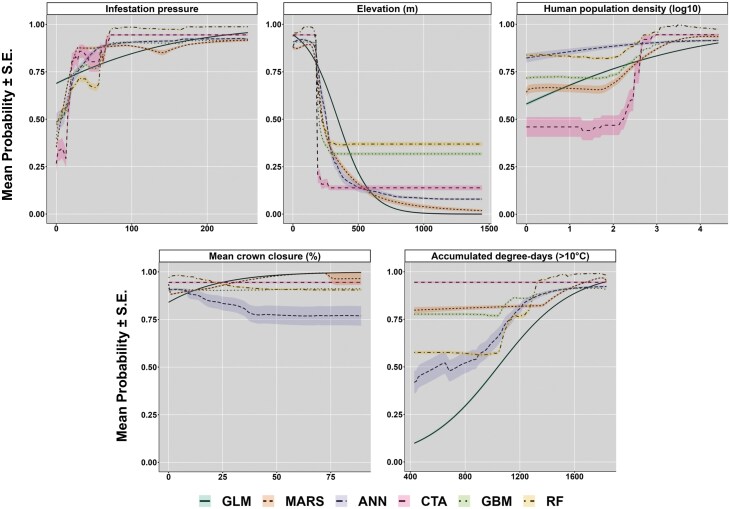
Mean predicted probabilities (±SE) of EAB presence as a function a given predictor, keeping the other variables fixed (at the median; at 0 for landcover) for 5 influential variables determined from VI analyses. The curves show mean probabilities for 30 runs per model (29 in case of ANN). GLM = generalized linear model; MARS = multiple adaptive regression splines; ANN = artificial neural networks; CTA = classification tree analysis; GBM = generalized boosting model; RF = random forest.

For different model types, mean probabilities of EAB presence as a function of changing values of a given predictor (keeping the other predictors fixed at the median, or at 0 for landcover), for 5 most influential variables determined through VI analyses are shown in Fig. 5. Overall, relative consistency in the trends was observed among the models for infestation pressure, elevation, and human population density (Fig. 5). The relationship between the presence of EAB and accumulated degree-days (>10 °C) was consistently positive except for the CTA models, where mean probabilities were high irrespective of the predictor values (Fig. 5). Differences among models were more apparent for mean crown closure, which showed opposing trends between regression-based models (GLM, MARS) versus ANN or RF models. CTA and GBM models suggested no effect of mean crown closure on the average probability of EAB presence.

**Fig. 6.**
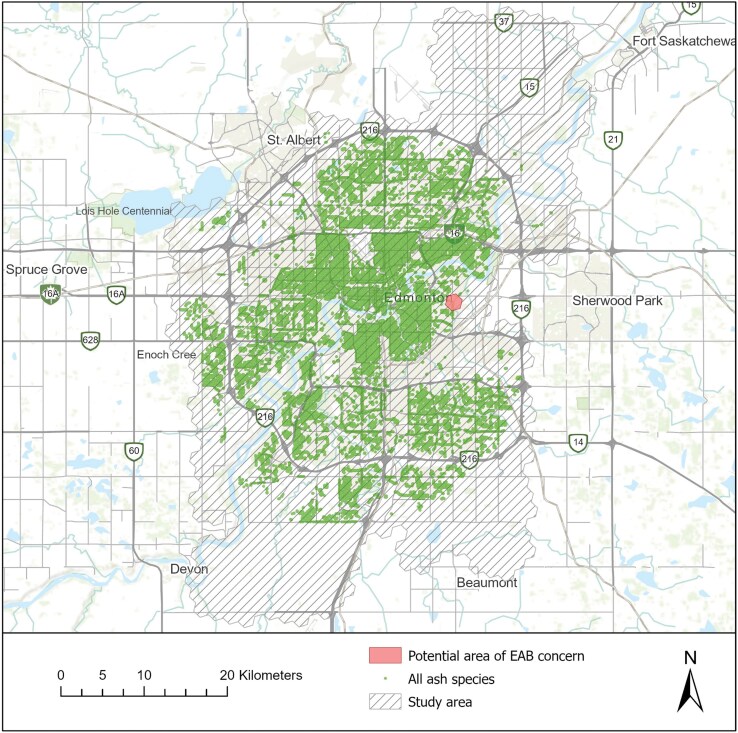
A map showing 1 potential area of EAB concern in the city of Edmonton, Alberta, Canada, based on predictions from Random Forest and ensemble models.

Our RF and ensemble model forecasts indicated that the City of Edmonton, Alberta, Canada, may be at a low, albeit non-negligible, risk of EAB infestation, with 1 RF model and 1 ensemble model (out of 30 each) identifying an area of concern (53.5371°, −113.4173°; Fig. 6).

## Discussion

This study compared several single and multivariable candidate models, using relevant predictors across 5 driver groups, to assess their goodness-of-fit to EAB presence–absence data from Canada. Another aim was to assess whether an ensemble of multiple different types of models provided a better performance compared with individual models. As expected, candidate models that included a combination of explanatory variables from different driver groups had greater support than those that included 1 or more explanatory variables from any particular driver group. We also found that an ensemble of different model types did not result in an improved performance compared with the RF algorithm, which was the best-performing overall. Elevation was found to be the most influential variable in predicting EAB occurrences, which has not been reported previously to our knowledge.

We studied the strength and direction of the relationships between EAB occurrences and the explanatory variables for single-variable and multivariable logistic regression models. Incorporating neighborhood infestation pressure improved the predictions, similar to studies on other mobile species, including EAB (Augustin et al. 1996, Sambaraju et al. 2012, Short et al. 2020, Ward et al. 2020). Adult EAB have high dispersal capability (Taylor et al. 2010), and combined with frequent, human-driven introduction events such as through transport of infested materials, the likelihood of finding new infestation foci around preexisting infestations is high (Short et al. 2020, Ward et al. 2020) if the surrounding landscapes do not create significant barriers to beetle movement. Local topography may be important to EAB success. For instance, after accounting for other factors in a multivariable logistic regression model, slope was positively associated with EAB infestations alluding to the potential importance of soil moisture and nutrient conditions on EAB occurrences. However, research to date provides only weak evidence that certain ash species under water deficit conditions are linked to better EAB fitness (Chakraborty et al. 2014, Rutledge and Arango-Velez 2017, Showalter et al. 2018). Proxy indicators of human-mediated spread of EAB (eg through firewood, etc.) such as human population density and transport pathways were positively associated with an EAB infestation. These observations agree with previous studies (Short et al. 2020, Ward et al. 2020) and are important to formulate ecological and economic impact assessments associated with protecting and managing ash tree resources in urban centers (Kovacs et al. 2010).

Relationships of certain host-related predictors with the likelihood of EAB presence were generally weak and variable. When used independently in single predictor logistic models and after accounting for spatiotemporal dependencies, only landcover class was a significant predictor of EAB presence. However, controlling for multiple other key factors, increasing crown closure was associated with an increased risk of EAB infestations. This goes counter to the expectation that the association would be negative given EAB’s preference for ash trees in sunny conditions over those in shaded areas (McCullough et al. 2009, Wang et al. 2010). This inconsistency indicates a weak relationship between crown closure and EAB presence, or suggests the influence of confounding factors such as targeted trapping in ash-dense, close canopy sites, variable densities of ash trees versus non-ash trees, and management measures such as tree removals and insecticide applications (Siegert et al. 2010, Ward et al. 2020). Also, our results suggest that EAB preferences for individual trees in sunny areas may not translate into a coarse-scale phenomena. Finally, urban areas were most associated with infestations which is generally the case with non-native woodborers transported via infested materials through human activity.

Warmer temperatures, resulting in higher accumulated degree-days, over the course of the year increased the likelihood of EAB presence. EAB lifecycle and flight behavior are influenced by temperature regimes (Duan et al. 2013, Fahrner et al. 2015). Higher temperatures can accelerate larval development and influence flight periods. The beginning, peak, and cessation of flight activity are related to accumulation of degree-days over 10 °C (Brown-Rytlewski and Wilson 2004, Poland et al. 2011). In Michigan, for example, adult emergence typically occurs in early summer between 230 and 260 DD_10_ (Brown-Rytlewski and Wilson 2004). Poland et al. (2011) reported peak EAB emergences between 800 and 1,200 accumulated degree-days over 10 °C and ~95% beetle captures between 1,327 and 1,580 degree-days in Michigan. However, since degree-day accumulations will vary by location and year, timing of adult emergence can correspondingly vary (Jones et al. 2020, Bohannon et al. 2022).

Decreasing coldest quarter mean temperature decreased the likelihood of EAB presence in single predictor models, while it was not significant in the final multivariable logistic model. This indicates that, after considering other factors, winter weather may not be a constraint to the spread of EAB. Indeed, a recent study by Duell et al. (2022) showed that EAB larvae and prepupae have more pronounced plasticity to cold temperatures than previously thought, with some surviving temperatures as low as −50 °C. Nevertheless, it is possible that extreme cold preceded by prolonged warm spells may cause serious mortality of overwintering EAB (Sobek-Swant et al. 2012a), which needs to be considered in future modeling efforts. Also, variables derived from stage-specific temperature thresholds for EAB growth should be considered in developing process-based models (Duan et al. 2013). In our study, we do not believe that omission of stage-specific temperature predictors would noticeably impact our modeling results because of the potential for high correlation among various temperature-based variables.

Multi-model comparisons are increasingly being adopted for different purposes including pest risk assessments, disease epidemiological modeling, exploring biological invasions, and studying species distributions under climate change across taxa (Sharma and Jackson 2008, Virkkala et al. 2010, Shabani et al. 2016, Sambaraju and Côté 2021, Herpin-Saunier et al. 2022). This approach allows modelers to check the consistency in predictions and VIs among the different models, and to combine best-quality models of different types via averaging or majority voting (Araújo and New 2007, Thuiller et al. 2024). “Ensembling” predictions from multiple types of models is expected to improve the predictions (Hao et al. 2020). In contrast, we found that the RF algorithm outperformed the ensemble model and all other individual modeling techniques. Crocker et al. (2010) used RF to study associations of EAB occurrences with landscape metrics and human population density and found the model to have high predictive accuracy. In other SDM studies, RF, GBM, and ANN have been shown to provide equivalent or even better performances than traditional statistical models, and even ensembles in some cases (Koo et al. 2018, Hao et al. 2020, Sambaraju and Côté 2021, Sharma et al. 2021). Certain underperforming models may have lowered the performance of the ensemble model in our study compared with RF. Nonetheless, the difference in TSS values between RF and ensemble models was quite small (2% to 3%). Finally, a single ANN model run failed in our multi-model analysis possibly due to computational issues during model training and/or due to the inability of the model to capture response–predictor relationships given the data sampled (Maier et al. 2023). We do not expect a single omission (out of 30 runs) to have any notable impact on the results.

Our comparative analysis provided the opportunity to assess the importance of variables and marginal response probabilities with changing values of 5 influential predictors (Fig. 5). In VI analysis, all model types tested in this study consistently showed that elevation, infestation pressure, accumulated degree-days > 10 °C, and human population density were important and informative predictors of EAB infestations. Elevation was the most important variable for predicting occurrences of EAB across the models tested. This is an important finding because we are unaware of any previous studies in North America that reported this association. However, we do note that elevation effects on EAB may be due to variations in temperature and precipitation, which have been considered in previous modeling efforts (Sobek-Swant et al. 2012b, Ward et al. 2020). Marginal response probability plots showed a relative consistency in the trends across most models for infestation pressure, elevation, human population density, and accumulated degree-days. However, inconsistencies among the models were more prominent for mean crown closure. These differences may be attributed to model structure, ability (or inability) of certain models to capture complex, nonlinear relationships, underlying distribution assumptions or lack thereof, and differences in parameters.

We identify certain limitations and caveats underlying our analysis that should be carefully considered while interpreting the results of this work, and offer some suggestions for improvements in future modeling studies:

1. EAB occurrence data: Our study was limited by the EAB data set that we used and considerations regarding the general biology and ecology of EAB. For instance, concerning the EAB data set, although we made a strong effort to gather and combine EAB survey data from as many sources as possible, other agencies or individuals would have likely had additional data that were not freely available or that we were not aware of during data collection. Inclusion of these points would have further improved the goodness-of-fit of predictions by further accurately characterizing the dispersal kernel.
2. A source of potential bias is introduced via regulatory or control measures that would restrict the movements of EAB through infested materials (eg firewood, nursery stock) beyond the target quarantine zones or influence its presence and spread potential within and beyond a region, respectively. Thus, the observed distribution of EAB may be considered a partial representation of a hypothetical full distribution when no constraints to EAB movements were to exist.
3. Detectability and sampling: Imperfect detection of EAB due to its cryptic nature of infestations (ie infested but asymptomatic trees), temporal evolution in survey methodologies used (eg visual surveys vs. traps), and variable sampling efforts over space and time introduce further uncertainty regarding some of the “true” absence observations and this will impact the dispersal kernel, and thus the modeling results. Future studies should consider combining survey information for a given study unit/grid cell over a longer time frame (eg 5-yr period) rather than using annual occurrence data in forecasting models.
4. Host maps: Another limitation concerns the maps of host-related predictors (crown closure, treed and vegetated, and broad-leaf percentages; Beaudoin et al. 2017) that we used. These maps are of relatively coarse spatial resolution (250 m) and have variable accuracy across Canada that would likely impact the modeling performances. Using more accurate maps of host-related predictors with higher (25 m) spatial resolution (Guindon et al. 2024) could improve model predictions.
5. Modeling approach: We chose logistic regression to assess individual relationships and screen predictors for multi-model analyses due to the ease of model interpretation, which is not the case with more complicated approaches (eg decision trees). However, logistic regression does not inherently capture influences of unmeasured factors or complex, nonlinear response–predictor relationships that, respectively, mixed effects models or advanced machine learning algorithms can do (Pinheiro and Bates 2006, Shmueli 2010). So, the various predictors considered important can change by the initial modeling approach used, although we expect that the main predictors (Figs. 4 and 5) will likely remain the same given the consistency across models. It is recommended that future studies examine alternative methodological approaches to assess consistency in response–predictor relationships and variables retained for developing predictive models.
6. Climate change impacts: We did not study the impacts of climate change on EAB distribution, which was beyond the scope of this study. There is a need for more detailed information on stage-specific growth rates and temperature tolerance profiles for EAB to develop accurate forecasting models. For instance, inclusion of physiological model-based outputs in correlative models of the kind that we developed would likely improve future forecasts of infestation risks under different climate change scenarios. These kinds of forecasting models and tools are urgently needed to combat the threat posed by non-native insects in general.

EAB continues to spread in North America and European Russia west into central Europe. Furthermore, continued interceptions of harmful non-native forest insects and their potential establishment/spread are a growing concern (Aukema et al. 2010). High-performing correlative models and process-based mechanistic models, as well as hybrid models combining the strengths of both of these approaches, are therefore needed for risk assessment and for management purposes. Our study contributes to this endeavor. For example, our model forecasts of possible EAB presence can inform decision-making regarding proactive detection surveys and understanding EAB’s spread potential. Although correlative models like those in this study using local data may not be readily transferable to new regions for risk assessments (eg Europe), they do provide important insights and a template for model development and evaluation that can be adopted for EAB and other invasive forest pests in those regions. From a practical recommendation standpoint, averaging across models and from a coarse-scale perspective when EAB is present on the landscape, our results suggest that ash-growing regions at low elevation areas (0 to 175 m), population densities of <1,000 persons per sq. km (as the presence probability increased steeply in this range and plateaued thereafter), and areas showing warming trends resulting in higher degree-day accumulations (~430 DD_10_ or higher) may need to be closely monitored for EAB infestations. A further understanding of mechanisms underlying EAB’s distributions within the core range and at the expanding range edges will help us gain insights into processes driving the spread and in the development of more accurate risk models. Finally, modeling exercises such as ours would hugely benefit from readily accessible georeferenced data on non-native pests and a broader range of predictor variables made available across large spatial extents. Collaboration among different institutional data holders is needed to build a publicly accessible and curated central repository of pest occurrence records.

## Supplementary Material

ieaf032_suppl_Supplementary_Figures_S1_Tables_S1_Appendixs_S1

## Data Availability

Data presented in this study are available from the corresponding author upon a reasonable request. Information on publicly available data sets is presented in the supplementary information section. Relevant R codes used in the modeling are available on Figshare (https://figshare.com/s/e7e1ff8bdf2faac9ca87, https://figshare.com/s/0310b2cace0a3dd0d917).
