# Supplementary material for: Multi-model assessments to characterize occurrences of emerald ash borer (Coleoptera: Buprestidae)
Source: J Insect Sci. 2025 Jun 3;25(3):18. doi: 10.1093/jisesa/ieaf032 (PMC12132038; doi:10.1093/jisesa/ieaf032)
Supplement: ieaf032_suppl_Supplementary_Figures_S1_Tables_S1_Appendixs_S1 [file ieaf032_suppl_supplementary_figures_s1_tables_s1_appendixs_s1.docx]

**Supplementary Table S1.** Links to web sources used in the development of models for the emerald ash borer.

| Data type | Source |
| --- | --- |
| Topography (Elevation, slope, and aspect) | Shuttle Radar Topographic Mission (SRTM) void filled DEM at 3-arcsecond (90 m) [https://doi.org/10.5066/F7F76B1X](https://doi.org/10.5066/F7F76B1X%20) (accessed June 2012) |
| Road data | <http://ftp.maps.canada.ca/pub/statcan_statcan/Road-networks_Reseau-routier/canadian_road_network/canadian_road_network_en.gdb.zip> (Statistics Canada, accessed 17 October 2018).  <https://www12.statcan.gc.ca/census-recensement/2011/geo/RNF-FRR/index-2011-eng.cfm?year=16> (Statistics Canada, 14 September, 2022).  <http://ftp.maps.canada.ca/pub/nrcan_rncan/vector/geobase_nrn_rrn/> (Natural Resources Canada, 14 September 2022). |
| Railway network data | <http://ftp.maps.canada.ca/pub/nrcan_rncan/vector/geobase_nrwn_rfn/> (Natural Resources Canada; accessed 14 September 2022) |
| Campgrounds | Camping Québec; ArcGIS online campground data: <https://www.arcgis.com/home/item.html?id=f3f33f5227fc422fae2d76b9082b7a80#overview> (accessed 14 September, 2022).  Manual collection of campground data using Google maps across Canada using provincial tourism websites, other online sources, and personal communications. |
| Federal and provincial parks | <https://open.canada.ca/data/en/dataset/9e1507cd-f25c-4c64-995b-6563bf9d65bd> (accessed 12 July, 2023)  Accessed 12 September, 2022:  <http://ftp.maps.canada.ca/pub/pc_pc/National-parks_Parc-national/national_parks_boundaries/>  <http://mli2.gov.mb.ca/adminbnd/index.html>  <http://www.snb.ca/geonb1/e/DC/catalogue-E.asp>  <https://www.arcgis.com/home/item.html?id=d1d705a4454a4bb1b95422ea74d6d2b2>  <https://www.albertaparks.ca/albertaparksca/library/downloadable-data-sets/>  <https://catalogue.data.gov.bc.ca/dataset/bc-parks-ecological-reserves-and-protected-areas>  <https://www.tcii.gov.nl.ca/parks/gis_data.html>  <https://www.arcgis.com/home/item.html?id=92357d38589a40d38b96a3d2fbe14e01>  <http://www.gov.pe.ca/gis/download.php3?name=prov_parks&file_format=SHP>  <https://www.arcgis.com/home/item.html?id=da0bb177d03c40a2931ebedffd8d4319>  <https://www.arcgis.com/home/item.html?id=0e8c9e43fbac4aca981c396c2ca1c94b> |
| Human population density | <http://cidportal.jrc.ec.europa.eu/ftp/jrc-opendata/GHSL/GHS_POP_GPW4_GLOBE_R2015A/GHS_POP_GPW42015_GLOBE_R2015A_54009_250/V1-0> (accessed 14 September, 2022)  <https://open.canada.ca/data/en/dataset/6715567e-2844-464a-9a13-fe76fc11da8f> (14 September, 2022) |
| Vegetation layers | <https://open.canada.ca/data/en/dataset/ec9e2659-1c29-4ddb-87a2-6aced147a990> (accessed 14 September, 2022) |
| Landcover | <https://open.canada.ca/data/en/dataset/39518dfa-bb8d-8a04-b36b-50b4310527a2> (accessed 4 March, 2024) |
| Weather data (BioSIM) | [https://apps-scf-cfs.nrcan.gc.ca/biosim](https://can01.safelinks.protection.outlook.com/?url=https%3A%2F%2Fapps-scf-cfs.nrcan.gc.ca%2Fbiosim&data=05%7C02%7Ckishan.sambaraju%40NRCan-RNCan.gc.ca%7C19a17cfd18fd484f773208dc3c93039c%7C05c95b3390ca49d5b644288b930b912b%7C0%7C0%7C638451849313080820%7CUnknown%7CTWFpbGZsb3d8eyJWIjoiMC4wLjAwMDAiLCJQIjoiV2luMzIiLCJBTiI6Ik1haWwiLCJXVCI6Mn0%3D%7C0%7C%7C%7C&sdata=C%2F3jd1xwYMskzOE%2BsXEU94y6IPhC9q4mIBrNQyZH6PA%3D&reserved=0) (accessed 4 March, 2024). |
| Edmonton ash tree distribution | <https://data.edmonton.ca/Environmental-Services/Trees/eecg-fc54/about_data> (accessed 18 July, 2024). |

**Supplementary Fig. S1.** Change in colonization potential of a target site [exp(-*d*/*a*)] based on distance (*d*) to a source population. The value of the term *a* changes the shape of the colonization potential-to-distance relationship.


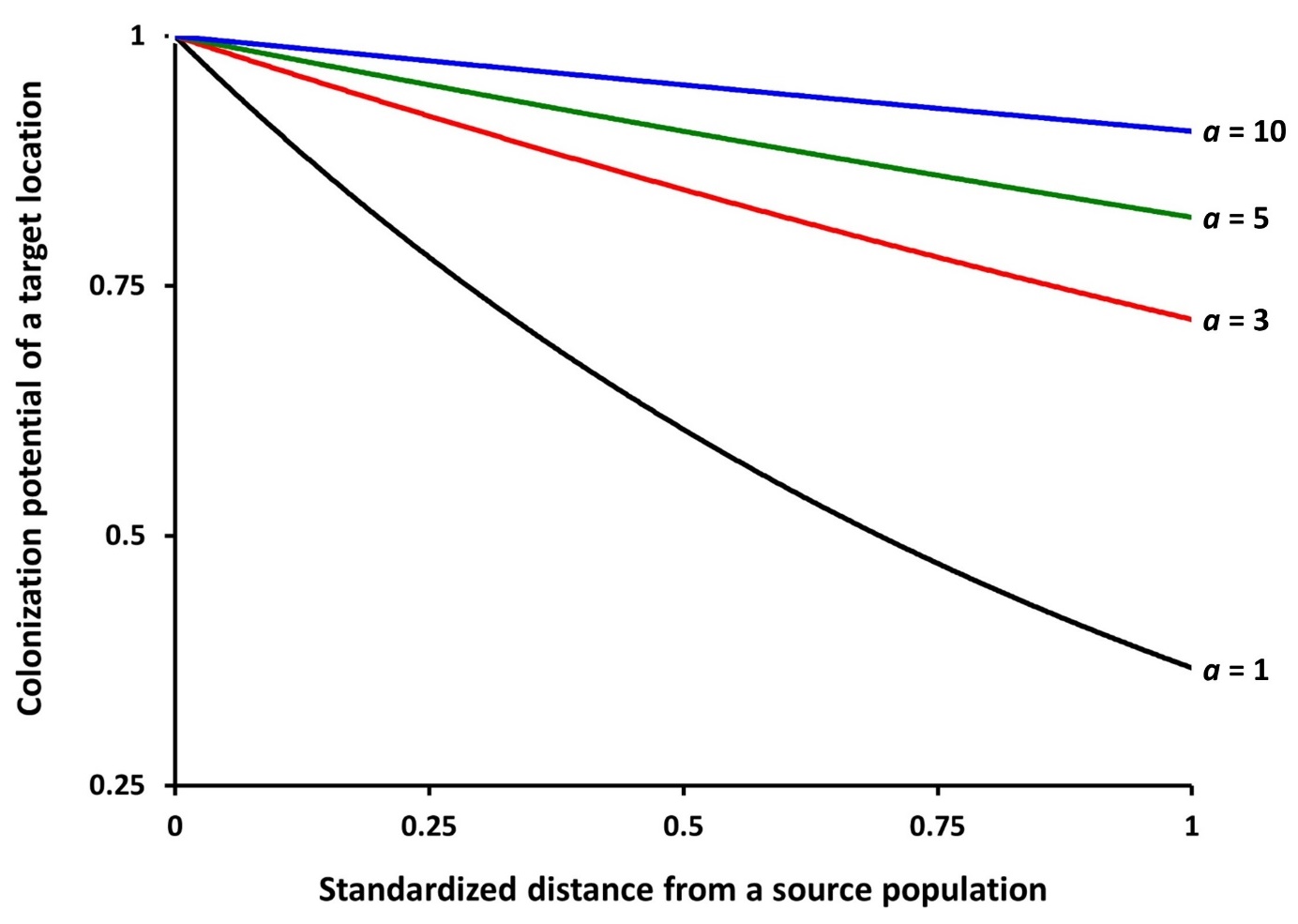


**Supplementary Appendix S1.** Parameterization of different model types used in multi-model assessments to predict presences of emerald ash borer in Canada. Description of the parameters and links for more details can be found in the documentation for *biomod2* (Thuiller et al. 2024).

GLM = list( type = 'simple',

interaction.level = 0,

myFormula = NULL,

test = 'BIC',

family = binomial(link = 'logit'),

mustart = 0.5,

control = glm.control(epsilon = 1e-08, maxit = 1000, trace = FALSE) ),

GBM = list( distribution = 'bernoulli',

n.trees = 2500,

interaction.depth = 7,

n.minobsinnode = 5,

shrinkage = 0.001,

bag.fraction = 0.5,

train.fraction = 1,

cv.folds = 3,

keep.data = FALSE,

verbose = FALSE,

perf.method = 'cv',

n.cores = 1),

CTA = list( method = 'class',

parms = 'default',

cost = NULL,

control = list(xval = 5, minbucket = 5, minsplit = 5, cp = 0.001, maxdepth = 25) ),

ANN = list( NbCV = 5,

size = NULL,

decay = NULL,

rang = 0.1,

maxit = 1000),

MARS = list( type = 'simple',

interaction.level = 0,

myFormula = NULL,

nk = NULL,

penalty = 2,

thresh = 0.001,

nprune = NULL,

pmethod = 'backward'),

RF = list( do.classif = TRUE,

ntree = 1000,

mtry = 'default',

sampsize = NULL,

nodesize = 5,

maxnodes = NULL))

**Reference**

Thuiller W, Georges D, Gueguen M et al. 2024. Package 'biomod2': Ensemble platform for species distribution modeling. R package version 4.2-5-2. https://cran.rproject.org/web/packages/biomod2/biomod2.pdf.
